# Molecular structure and characteristics of phytoglycogen, glycogen and amylopectin subjected to mild acid hydrolysis

**DOI:** 10.1038/s41538-023-00201-6

**Published:** 2023-06-08

**Authors:** Bo Pan, Ningjing Zhao, Qiuqi Xie, Yungao Li, Bruce R. Hamaker, Ming Miao

**Affiliations:** 1 State Key Laboratory of Food Science & Technology, Jiangnan University, 1800 Lihu Avenue, Wuxi, Jiangsu 214122 P. R. China; 2 Whistler Center for Carbohydrate Research and Department of Food Science, Purdue University, 745 Agriculture Mall Drive, West Lafayette, IN 47907-2009 USA

**Keywords:** Polysaccharides, Dietary carbohydrates

## Abstract

The structure and properties of phytoglycogen and glycogen subjected to acid hydrolysis was investigated using amylopectin as a reference. The degradation took place in two stages and the degree of hydrolysis was in the following order: amylopectin > phytoglycogen > glycogen. Upon acid hydrolysis, the molar mass distribution of phytoglycogen or glycogen gradually shifted to the smaller and broadening distribution region, whereas the distribution of amyopectin changed from bimodal to monomodal shape. The kinetic rate constant for depolymerization of phytoglycogen, amylopectin, and glycogen were 3.45 × 10^−5^/s, 6.13 × 10^−5^/s, and 0.96 × 10^−5^/s, respectively. The acid-treated sample had the smaller particle radius, lower percentage of α-1,6 linkage as well as higher rapidly digestible starch fractions. The depolymerization models were built to interpret the structural differences of glucose polymer during acid treatment, which would provide guideline to improve the structure understanding and precise application of branched glucan with desired properties.

## Introduction

Polysaccharide are one kind of widespread organic macromolecule in nature and produce in green plants by photosynthesis and serve as the essential structural and energy-reserve constituents of all living things^1^. Polysaccharides consist of tens to hundreds to several thousand monosaccharide units linked together, and vary considerably in the molecular size and monosaccharide compositions for the structural complexity^2,3^. Primary sources of most common polysaccharides are cellulose and starch, which are extensively used in a wide variety of industrial applications, such as food, pharmaceuticals, agriculture, textiles, and paper products^1^. In recent years, there has been increasing interest in nano-scale water-soluble polysaccharides as an important class of biomaterial, due to their potential functionalities and bioactivities^4–6^. Phytoglycogen as amylopectin analogue is a dendrimer-like starch-related nano-particle naturally occurring in various plants, including the sugary-1 mutants of maize, rice, sorghum, barley as well as Arabidopsis^4,6^. Phytoglycogen was observed as the spherical shape with the nano-size, different from the worm-like shape for amylopectin with larger size^7^. From the structural perspective, phytoglycogen contains only one reducing end in the core and a large number of small glucan chains attached to one another via α-1,4 or α-1,6 linkages. According to recent literatures^8–11^, there were two typical models for glycogen particle conformation with either sharp and dense or loose and diffuse for tier model or hairy particles model, respectively. For tier model, the ρ increased from the core towards the outer surface of the particulate and the number of tiers had a limit of 12, resulting in the steric hindrance for chain growth at the periphery. For the hairy particles model, the density profile is uniform within the particle, opposite to that predicted by the tier model as determined using pyrene excitation fluorescence^9^, small angle neutron scattering^10^, and computer simulations^11^. Moreover, glycogen has been proposed to bear a structural resemblance to phytoglycogen in animal tissue involved in glucose regulation^3^. Glycogen is composed of single molecular spheres (β-particles with 100 nm in diameter) and aggregated spheres of smaller particles (α-particles with 20 nm in diameter)^12^. Recent studies highlighted phytoglycogen or glycogen have been developed for use as the novel encapsulation and release-delivery platforms^13–16^. The hyper-branched particulate structure of glucose polymer determines the solubilisation, interface function and delivery property^5,13,15^, which in turn affect the nutritional characteristics of functional foods. Thus, the understanding of the structure changes during modification or processing is a vital issue to design tailor-made products.

Acid degradation reactions are of great interest in improving the understanding of fine structure and properties of polymers^17–19^. For example, acid hydrolysis was considered to be an important chemical modification for revealing the microstructure of starch granules and modifying their functional properties^18,20,21^. It is all known that acid degradation preferably starts from amorphous region in the growth rings of starch granule, followed by a slow concomitant hydrolysis of semi-crystalline alternating regions^18,22^. At the level of fine chain structure of starch, there were two residual chain populations with peak maxima of DP 15 and 25 after prolonged acid degradation, which was originated from the singly double helices with amylopectin A and B1 chains^20^. An increase of medium chain fragments of amylopectin (DP 13-33) with the reduction of long (DP > 33) and short (DP < 13) chains was observed for acid hydrolysis of waxy maize starch^18^. Moreover, the functional properties of starch are altered significantly when the acid hydrolysis progresses, which can be used for different industrial applications, such as thin-boiling starches, nano-crystals and resistant dextrins^1,23,24^. The functional properties of starch can be tuned through chemical modification and acid hydrolysis is a simple approach for polysaccharides modification through breaking glycosidic bonds. The amylopectin analogue including phytoglycogen and glycogen exists as small nanoparticles with a different chain architecture and smaller molecular size, however, there is relatively little information pertaining to the mild acid degradation on their structure of nanoparticles^19,25^, and the related hydrolysis mechanisms on affecting properties have not been studied in detail. The goal of this work is to understand how hydrochloric acid hydrolysis can fragment phytoglycogen and glycogen and alter their particulate structure using amylopectin as a reference, and how acid-induced bond cleavage could in turn affect the functionalities. The results would provide important guideline for improving the structure understanding and the precise application of partial acid-treated glucose polymer in the development of novel biomaterial with desired properties.

## Results and Discussion

### Structural comparison of native glucose polymers

The structural differences between three substrates were measured using SEC-MALLS-RI. As shown in Table 1, Mw of phytoglycogen, amylopectin and glycogen as the starting materials were 2.14 × 10^7^, 3.74 × 10^7^ and 0.53 × 10^7^ g/mol, and R_z_ were 43.1, 167.8 and 29.4 nm, respectively. Both M_w_ and R_z_ were in the following order: amylopectin > phytoglycogen > glycogen. The M_w_ increased as the R_z_ increased for different sources of glucose polymer, indicating a positive relationship between M_w_ and R_z_. M_w_ of phytoglycogen was lower than that of amylopectin, due to the absence of starch debranching enzyme in starch biosynthetic in cereal endosperm^1^. Some previous literatures indicate that amylopectin is composed of linear chains of α-1,4-D-glucose units connected through α-1,6 linkages (5-6%), with an average chain length of DP 13-24^1^, whereas phytoglycogen is the amylopectin analogue in the endosperms from sugary-1 mutations of cereal grains with decreased activity of isoamylase-type starch debranching enzyme, forming linear chains with average chain length of DP 10-12 and 7-10% branch points^4,7^. Also, the ρ of phytoglycogen was approximately 10-fold than that of amylopectin^26^. Glycogen depicts the similar structural feature of phytoglycogen, however, there are two types of structural subunits known as α and β particles and larger clusters of α particles in a cauliflower-like structure is composed of small β particles^12^. Moreover, the dispersity (D) was used as a measure of the breadth of the molar mass distribution and D were in the following order: amylopectin (2.3) > glycogen (1.5) > phytoglycogen (1.1), which indicated that there was only monodisperse phytoglycogen arranged in chains of equal radius. Similar observations for phytoglycogen from different sugary maize cultivars have been reported by Miao and coworkers^4^.

**Table 1:** Molar mass and radius of gyration of native phytoglycogen, amylopectin and glycogen.

Sample	Mn (×10^7^ g/mol)	Mw (×10^7 ^g/mol)	Mz (×10^7 ^g/mol)	D	Rz (nm)
Phytoglycogen	1.89 ± 0.05	2.14 ± 0.01	2.66 ± 0.07	1.1 ± 0.1	43.1 ± 0.7
Amylopectin	3.72 ± 0.10	8.37 ± 0.06	8.46 ± 0.04	2.3 ± 0.2	167.8 ± 2.3
Glycogen	0.36 ± 0.02	0.53 ± 0.05	0.75 ± 0.11	1.5 ± 0.1	29.4 ± 0.6

*Mn* number-average molar mass, *Mw* weight-average molar mass, *Mz* z-average molar mass, *D, dispersity* D was calculated as Mw/Mn, *Rz* z-root mean square radius of gyration.

### Acid hydrolysis

**Fig. 1:**
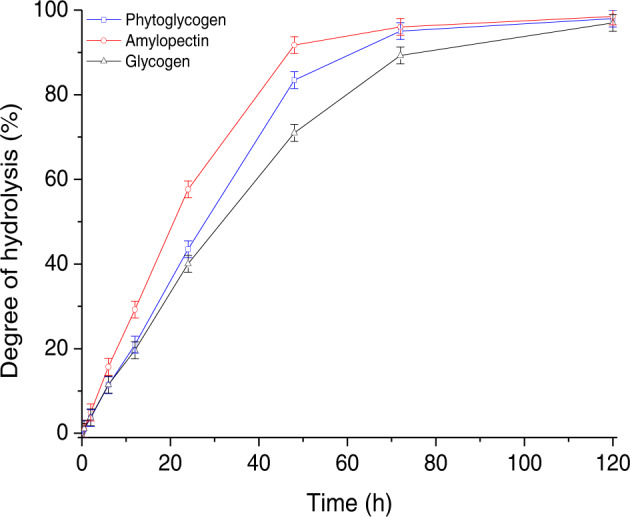
Hydrolysis profiles of acid-treated phytoglycogen, amylopectin and glycogen over time at 50 C. Error bars for three replicates indicated s.d.

The hydrolysis profiles of acid-treated phytoglycogen, amylopectin and glycogen over 120 h are presented in Fig. 1. The time course of acid degradation of substrate took place in two stages. In the rapid degradation stage (approximately 0-48 h), the degree of hydrolysis increased substantially up to higher than 70% due to rapidly degradation of glucose polymer, whereas the degree of hydrolysis of substrate incrementally increased to the limit values of approximately 100% at 120 h for the later slow degradation stage. The similar trends were observed for three polymers, however, the degree of hydrolysis was in the following order under the same reaction conditions: amylopectin > phytoglycogen > glycogen, which may be related to the different fine structure of substrate^18,22,25^. The differences in the spatial dendritic architecture including chain length, molar mass, particle radius and molecular density of polymers might be attributed to the variation in acid hydrolysis profiles^18,21^.

Combined chromatogram data from different substrate (Table 1), the degree of hydrolysis increased by the increased molecular radius of glucose polymer. Sullivan and coworkers also reported that the larger particles in glycogen appeared to be degraded significantly more than the smaller one, suggesting that the α-1,4 glycosidic linkage resistance to acid hydrolysis compared to the protein-like links between particles^12^. Moreover, the degradation rate of the terminal α-1,4 linkage was faster than the other α-1,4 linkage on the glucan chain, whereas α-1,4 linkage was hydrolyzed seven times as fast as the α-1,6 linkage at room temperature^18^. On the basis of the above data, five types of acid-treated samples (phytoglycogen, amylopectin or glycogen subjected to acid hydrolysis for 5 min, 30 min, 2 h, 6 h, or 12 h, respectively) were selected to elucidate the kinetic and structure in subsequent experiments.

### Molar mass and radius of gyration analysis

**Fig. 2:**
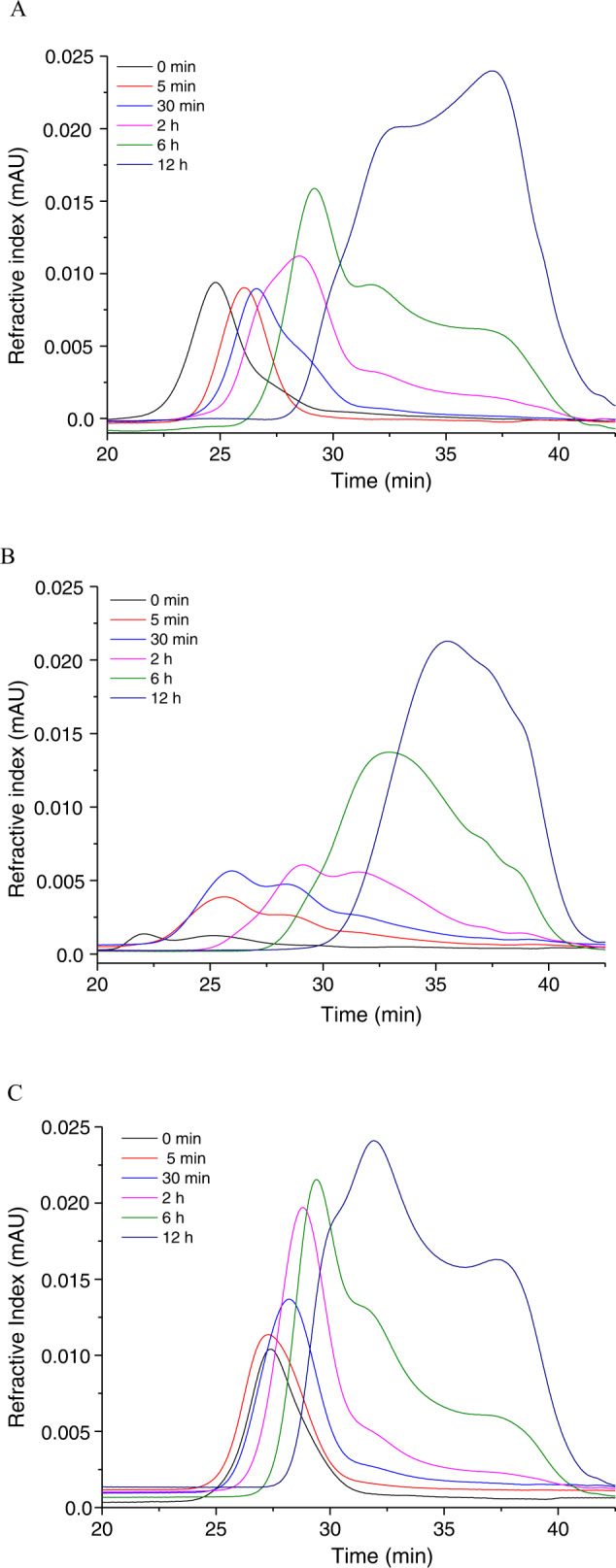
Variation of the molar mass distribution in the course of acid degradation. **A** Phytoglycogen, **B** amylopectin and **C** glycogen.

As shown in Fig. 2A, the chromatogram of phytoglycogen appeared as a monomodal and relatively narrow peak, indicating a homogenous polysaccharide as suggested by our previous study^4^. Also, the small shoulder at larger elution time was observed for native phytoglycogen, which nearly disappeared during acid hydrolysis, indicating the presence of some smaller particles as reported by Powell and coworkers^27^. They suggested that the small molecular-sized residual substances may be degraded molecules arising from the extraction treatment, as well as the contaminants or residual substances not removed by the initial extraction techniques. Upon acid hydrolysis of phytoglycogen, the distribution gradually shifted to the smaller molar mass distribution region with significantly broadening. It was noteworthy that a shoulder appeared in the distribution curve in the early stage of acid hydrolysis (after approximately 30 min), due to the formation of small fractions. The bimodal distribution curve appeared after hydrolysis of 12 h, revealing the heterodisperse characteristic of the treated phytoglycogen. For the acid hydrolysis of amylopectin, in contrast, the distribution changed from bi-modal to mono-modal shape (Fig. 2B), which indicated that the larger fragments of amylopectin would be preferentially degraded to small ones. Li and Hu reported that the first-order kinetics models were applied to fit the evolution curve of starch chain-length and molecular size by acid hydrolysis treatment and the fast hydrolysis phase involved degradation of amylopectin long intra-cluster branches^20^. This was related with the hydrolysis mechanisms: hydrogen ions more readily hydrolyzes the 2 or 3 glucose units away from the branching points of amylopectin long intra-cluster branches, and amylopectin short intra-cluster branches connecting the two neighboring double helices are less preferable for the acid hydrolysis. Wang and coworkers reported that small peaks (DP < 12) and peak shoulders (DP > 12) were detected between the two main peaks of DP 13-15 and 25-27, which were suggested to arise from the branched amylopectin after acid degradation of pea starc^22^. Moreover, Powell et al. found that acid degradation occurred uniformly throughout the particulates and larger molecules degraded more quickly^19^. As shown in Fig. 2C for the acid degradation of glycogen, a broader eluting peak was observed after 5 min of acid hydrolysis, suggesting that glycogen was more heterogeneous and a fraction of larger particles was hydrolyzed into smaller particle during acid degradation, which was consistent with the previous observations^19^.

**Fig. 3:**
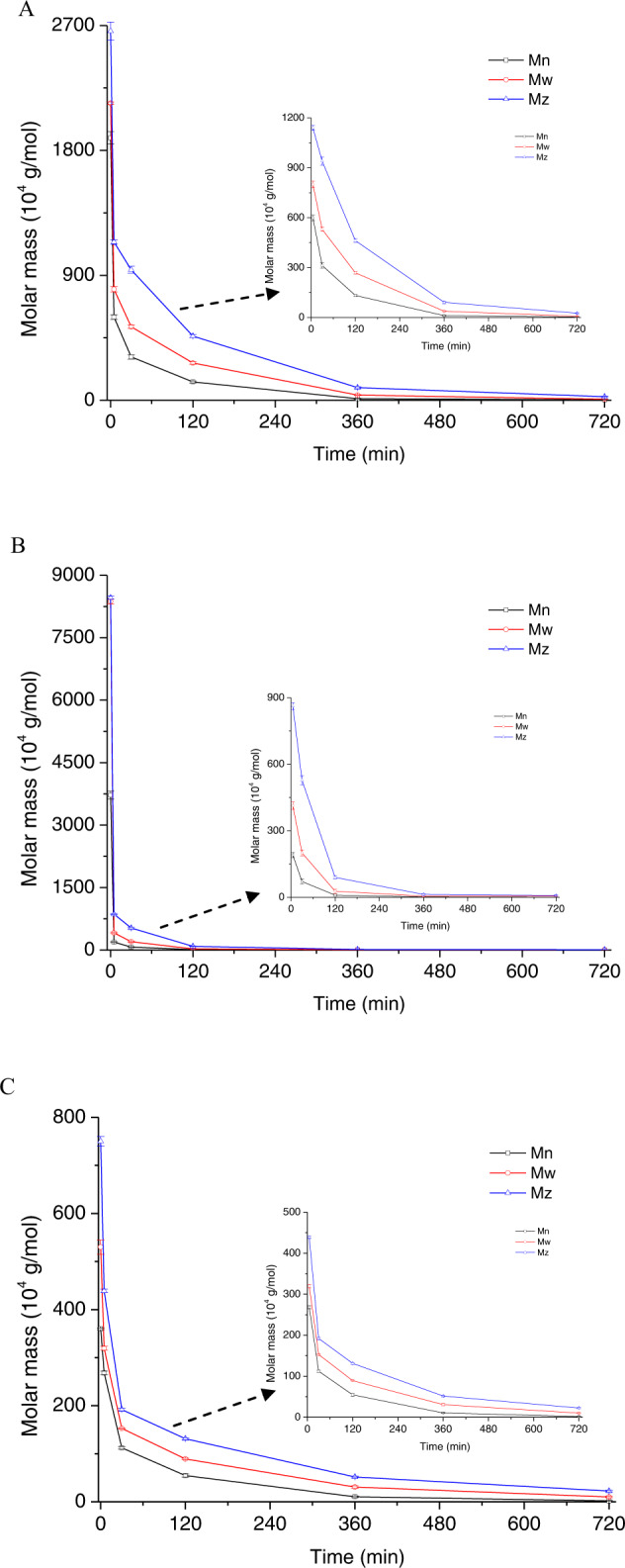
Variation of different average molar mass (Mn, Mw and Mz) with acid-treated time. **A** Phytoglycogen, **B** amylopectin and **C** glycogen. Error bars for three replicates indicated s.d.

Moreover, the variation of different average molar mass with acid treated time is shown in Fig. 3. A significant reduction in molar mass was observed during the course of acid degradation of Mn, Mw and Mz with the shifting maximum peak for the refractive index signal over time. Especially, a great reduction in molar mass was observed for the first 5 minutes of hydrolysis, following by a much more gradual reduction at longer hydrolysis times, and the molar mass of amylopectin decreased much more than for that of phytoglycogen or glycogen. This dependence of the molar mass on hydrolysis time was attributed to the acid hydrolysis of less dense regions in the amylopectin that was more accessible to the acid molecules, whereas the hydrolysis occurred more slowly within the denser regions of phytoglycogen or glycogen as described by Shamana and Dutcher^22^. The change of Mn was directly associated with the rate of reaction, however, there was a log relationship between molar mass and rate of reaction. Noticeably, Mn decreased more rapidly than Mw, whereas Mw decreased more rapidly than Mz in the early stages of hydrolysis. The greater degradation for amylopectin in the initial stage of acid hydrolysis was observed from the molar mass change of profile, compared with the phytoglycogen or glycogen as shown in Fig. 3. This might be related with the molecular chain conformation difference among the three substrates, resulting in varying small fragments (Rz >10 nm) during acid hydrolysis^25^.

### Kinetic modeling for depolymerization

To investigate the depolymerization kinetic of acid hydrolysis, the mathematical model was used to reflect the linkage cleavage of polymer by measuring the molar mass distribution with reaction time. As shown in Table 2, ρ of phytoglycogen, amylopectin and glycogen were 266.4, 17.7 and 209.4 g/mol·nm^3^, respectively, which indicated that either phytoglycogen or glycogen had more tightly packed rigid structure with greater dispersed density, compared to the amylopectin with long chain length for the relatively flexible structure^4^. After acid reaction, the value of ρ dropped substantially in the first 6 h, especially for phytoglycogen and glycogen. During the initial 5 min of acid treated glycogen, ρ was reduced to approach a half (from 209.4 to 107.4 g/mol·nm^3^), due to the formation of smaller particles (e.g. β particles or other degradation products) from glycogen degradation. This might be related with the chemical complexes exist between glycogen and binding protein under the “crowding-assembly” model^19^. Prats et al. reported glycogen as a proteoglucan has three structural types that termed α-granule, β-granule and γ-particle^28^. The α-granules are mainly found in liver and appear as aggregates of β-granules in a rosette-like pattern, while β-granules with molar mass of 10^6^-10^7^ Da have reported to exist in muscle with individual spherical structure containing several γ-particles. Also, the β particles have diameters varying between 10 nm and 50 nm, whereas the α particles have diameters as large as 300 nm, in a tetramer structure composed of small β particles^12^. It was suggested that the binding protein in glycogen was hydrolyzed faster than the glycosidic bonds under the acidic condition. A similar phenomenon was confirmed in our study as depicted in Fig. 3 and Table 2.

**Table 2:** Determination of the fine structure and kinetics of degradation products from phytoglycogen, amylopectin and glycogen over time.

Time (min)	Phytoglycogen					Amylopectin					Glycogen				
	Mw	Rz	ρ	D	CDR	Mw	Rz	ρ	D	CDR	Mw	Rz	ρ	D	CDR
0	2140.0	43.1	266.4	1.1	1.1	8370.0	128.8	17.7	2.3	1.1	530.1	29.4	209.4	1.5	0.9
5	799.8	32.7	228.9	1.5	0.9	413.3	62.1	17.2	2.2	0.8	319.5	30.9	107.4	1.2	0.6
30	529.9	34.2	132.5	1.7	1.1	200.2	53.5	13.1	2.8	0.8	152.9	25.7	89.7	1.4	0.4
120	268.4	47.8	24.6	2.0	1.1	27.3	33.7	7.1	2.8	0.8	89.2	37.7	16.6	1.6	0.2
360	37.3	35.8	8.1	2.4	1.7	5.5	29.2	2.2	3.0	1.3	30.8	29.2	12.4	2.9	1.4
720	7.5	21.1	0.8	2.8	2.3	4.9	23.6	3.7	12.4	7.4	10.2	21.8	9.8	5.0	0.8
k	3.45					6.13					0.96				

Mw (×10^4^ g/mol), weight-average molar mass; Rz (nm), z-root mean square radius of gyration; ρ (g/mol·nm^3^), molecular density; D, dispersity; CDR, combined dispersity ratio; k (×10^-5^/s), rate constant.ρ was calculated as Mw/Rz^3^. D was calculated as Mw/Mn. CDR was calculated as Mw^2^/MnMz.

D was defined as the broadness of the molar mass distribution and showed an increase with time, due to the formation of lower-size fragments, as depicted in the Fig. 2. Phytoglycogen had a monomodal distribution referring to the native particles, and D increased rapidly from 1.1 to 2.8 during the course of acid reaction. Shamana and Dutcher also reported that acid hydrolysis of phytoglycogen nanoparticles produced a decrease in the size, as well as an increase in polydispersity^25^. The similar trends were observed for glycogen and amylopectin, due to diversified structural products after acid degradation process. Significantly, D of glycogen decreased from 1.5 to 1.2 during the 5 min of acid treatment, which indicated that the obtained monodisperse smaller particle was from decreasing the larger size particles. The combined dispersity ratio (CDR) was unity for a log-normal molar mass and the enrichment in the higher weight region led to lower value of CDR and vice versa^29^. The original molar mass distribution was approximated by a lognormal curve with good accuracy (CDR at closer to 1). The CDR values for the degradation of the narrow phytoglycogen showed an increase with hydrolysis time.

**Fig. 4:**
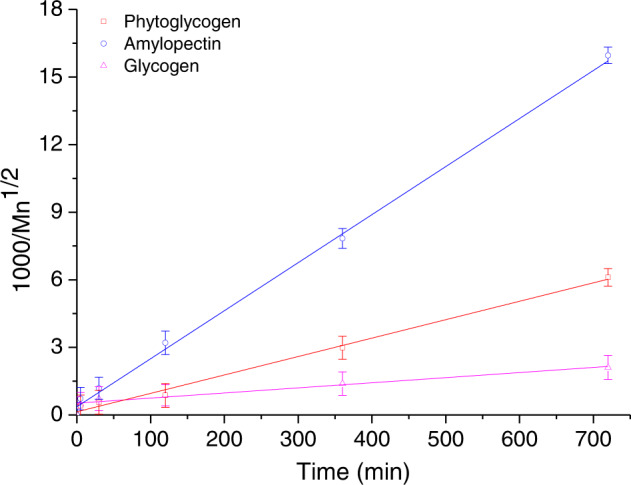
The number average molar mass as a function of reaction time. Error bars for three replicates indicated s.d.

Based on the simplified equation and experimental data, the value of α indicated the dependence of the reactivity of the polymer on the molar mass, and has been calculated to be 1/2 according to the previous literature^29^. A plot of 1000/Mn^1/2^ versus reaction time gave a straight line as shown in Fig. 4. The value of k for phytoglycogen, amylopectin, and glycogen were calculated from the slopes to be 3.45 × 10^-5^, 6.13 × 10^-5^, and 0.96 × 10^-5^ s^-1^, respectively. According to the previous studies^14,16^, the glucose polymer with a high molar mass was more stable to acid degradation than smaller fragments, and linkages near the end of polymer chain had a higher reactivity than those at the center. Moreover, the branched structure played a key role on the reactivity of polymer: more branched implied more rigid macromolecules, which suggested the chains formed highly branched and packed dendritic structure for the higher integrity of phytoglycogen or glycogen, compared to amylopectin with relatively flexible structure and long internal chains^4,26^. Therefore, the above results might indicate that the reactivity of the linkages changes due to the branched-chain composition, since the mobility of the chain was in the following order: amylopectin > phytoglycogen ≈ glycogen. However, further studies of branch-chain lengths and glycosidic linkages of substrates, as well as the acid concentration need to be conducted to address this issue, which certainly would help us to understand the fine structure in depth.

### Particle radius distribution analysis

**Fig. 5:**
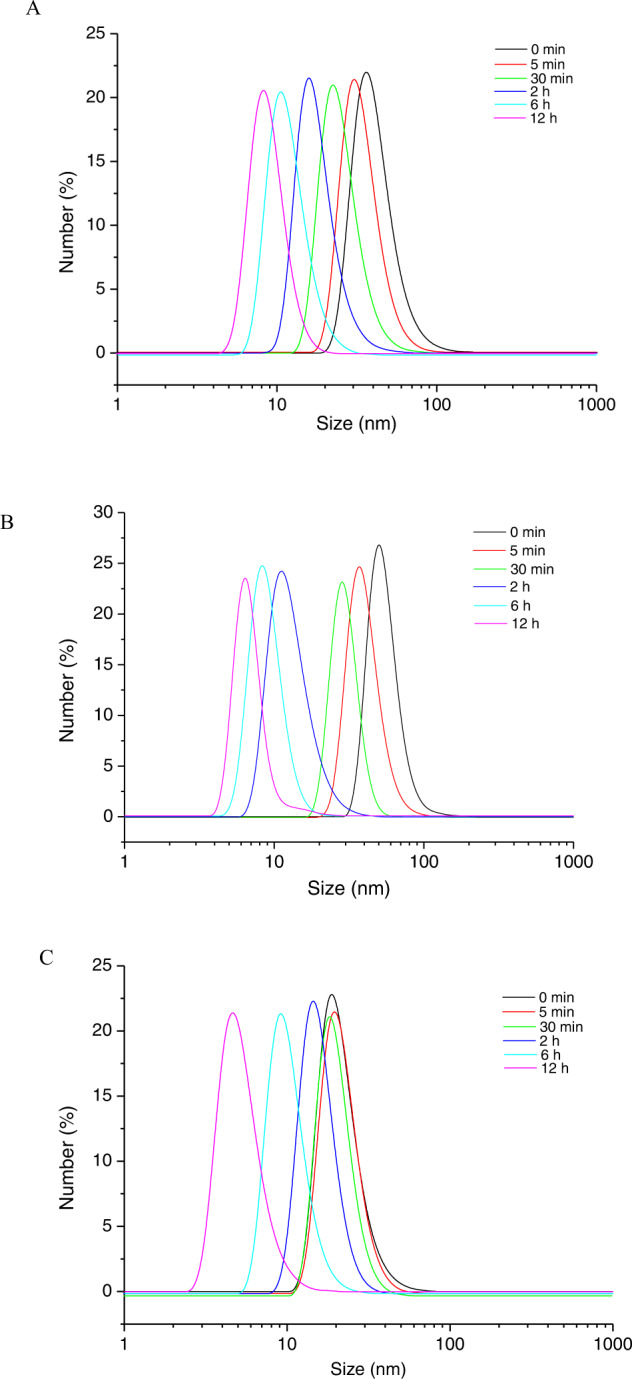
Particle size distributions of acid-treated sample over time. **A** Phytoglycogen, **B** amylopectin and **C** glycogen.

The particle radius distributions of acid-treated phytoglycogen, amylopectin and glycogen are shown in Fig. 5. The particle radius distributions of native phytoglycogen, amylopectin, and glycogen have been observed to exhibit the ranges of 25-122, 18-70, and 13-60 nm, respectively. The average particle radius was in the following order: amylopectin > phytoglycogen > glycogen. After acid hydrolysis, the distribution gradually shifted to the smaller particle radius distribution. For instance, the particle radius distributions were 5-18, 2-10, and 3-15 nm for acid-treated phytoglycogen, amylopectin, and glycogen after 720 min of hydrolysis, respectively, which was comparable with the obtained molar mass distributions as shown in Fig. 2. More specifically, there was a little narrower peak observed for the acid-treated glycogen with 5 min reaction, compared to the native glycogen, which was in accord with the results of D in Table 2. This was suggested that the formation of smaller particles (mainly β particles) in the early stage of acid degradation due to binding protein hydrolysis in glycogen^19^.

### ^1^H-NMR analysis

To further characterize the structural properties of acid-treated phytoglycogen, amylopectin and glycogen, their concentrations of α-1,4 and α-1,6 bonds were analyzed using ^1^H NMR spectroscopy. This approach can be used to determine the degree of branching of glucan dendrimer polymer, including phytoglycogen, glycogen and amylopectin. As shown in Table 3, the percentage of α-1,6 linkages of native phytoglycogen, amylopectin and glycogen were 7.6%, 5.2% and 6.5%, respectively, which indicated that the following order of phytoglycogen > glycogen > amylopectin. Similar observations for glucan dendrimer polymers have been reported by BeMiller and Whistler^1^. According to the previous studies^4,30^, phytoglycogen has been proposed to bear a resemblance to the glycogen isolated from animal sources. Phytoglycogen is made up of α-1,4-linked glucose units, forming linear chains with 7-10% branch points introduced by α-1,6 linkages that are relatively evenly distributed throughout the particles, and is primarily composed of internal short B-chains attached by external short B- and/or A-chains, with no long B chains^7^. Also, Yun and Matheson reported that glycogen has A:B chain ratio (where A chains contain only α-1,4 linkages and B chains also contain α-1,6 linkages) of < 1 and amylopectin ratios of > 1, which led to average frequencies of substitution of B chains over the whole molecule of < 2 for the glycogens and > 2 for the amylopectins^31^. After acid hydrolysis, the ^1^H NMR spectra showed an decrease of α-1,6 linkages in the acid-treated samples, which was related with the acid hydrolysis of glucosidic linkages in glucan chain. Especially, greater degradation was observed for phytoglycogen from 7.6% to 2.3% during acid hydrolysis course of 720 min, which might be due to larger particle radius of phytoglycogen as shown in Fig. 5. The branched structure of glucan polymer played a key role on the acid degradation: more radius implies larger surface, resulting in rapid hydrolysis of glucan chain with more time, comparable with the obtained ρ as shown in Table 2. Moreover, there was no change for the percentage of α-1,6 linkages in glycogen in the first 5 min, which might be related with the special aggregation structure of glycogen containing degradable protein as described by Tan and coworkers^32^. The above results suggest that mild acid acted on glucan chains and produced sample with shorter branch chains and less α-1,6 linkages.

**Table 3:** Concentrations of α-1,6 linkages of acid-treated phytoglycogen, amylopectin and glycogen.

Time (min)	Percentage of α-1,6 linkages (%)		
	Phytoglycogen	Amylopectin	Glycogen
0	7.6 ± 0.3	5.2 ± 0.5	6.5 ± 0.5
5	7.3 ± 0.5	4.8 ± 0.1	6.5 ± 1.0
120	5.8 ± 0.2	3.7 ± 0.2	5.9 ± 0.2
720	2.3 ± 0.6	1.9 ± 0.3	2.8 ± 0.2

### In-vitro digestion

Table 4 shows the enzymatic digestibility of acid-treated phytoglycogen, amylopectin and glycogen when subjected to the Englyst assay. According to the rate and extent of digestibility, the glucose polymer is divided into RDS, SDS and RS, which are the three consecutive nutritional fractions divided by reaction time and related to the physiological effect of processed food after consumption^33,34^. The results showed that RDS, SDS and RS were 74.7%, 12.2%, and 13.1%, 72.4%, 10.7%, and 16.8%, 69.9%, 12.9%, and 17.2% for native phytoglycogen, amylopectin, and glycogen, respectively, consistent with previous studies^4,7^. It has been reported that the enzymatic digestibility of glucan is influenced by source, particle radius, ratio of α-1,4 to α-1,6 bonds, molar mass and branch chain length^33,34^. This suggests that the total value for RDS and SDS of amylopectin was lower than that of phytoglycogen or glycogen, which was due to its lower α-1,6 bonds and longer branch chain length as well as larger particle radius. Pazur and Ando observed that hydrolysis of α-1,6 bond of glucose polymer was the rate-limiting step for the amylolytic degradation^35^, which revealed that the enzymatic hydrolysis rate of α-1,6 bond was approximately 30 folds lower than that of α-1,4 linkage. Also, there was a parabolic relationship between amylopectin molecular structure and SDS, which meant amylopectin with a higher amount of either short chains (DP < 13) or long chains (DP ≥ 13) had a higher content of SDS^36,37^. As illustrated in Table 4, the percentage of RDS was significantly increased in all of acid-treated samples, accompanying with decreasing the content of SDS. After acid hydrolysis, RS were composed of slightly difference, except for amylopectin with 720 min treatment, which might be attributed to the difference of branching patterns. Thus, the fine structure of glucose polymer substrate influencing the in vitro glucose release was a crucial factor for enzymatic digestibility, and the treated sample with more branch points, shorter chains and smaller radius had a higher SDS and RS content. According to the recent review of Miao and Hamaker^33^, the major intrinsic factors affecting starch digestibility include the botanical source, granule size, crystal architecture, ratio of amylose to amylopectin, amylopectin fine structure, and surface and interior characteristics of the starch granule. The smaller size of glucan samples have higher amylolysis rates than the larger counterparts, which is related with the increased surface-to-volume ratio of smaller particle for enzyme binding to the substrate, leading to a proportional increase in digestibility for acid-treated amylopectin. Also, there was a higher proportion of α-1,6 linkages in starch molecules, which are less efficiently degraded by amylase, resulting in low digestion properties. For the acid-treated glycogen, RS content increased by the increased hydrolysis time, which might be related with more α-1,6 linkages and larger particle size as well as some small product molecules with amylase inhibitory activity. Further studies need to be conducted to address this issue, which certainly would help us to understand the more resistant fractions of acid-treated glycogen in depth.

**Table 4:** In vitro digestion of acid-treated phytoglycogen, amylopectin and glycogen.

Time (min)	Phytoglycogen			Amylopectin			Glycogen		
	RDS(%)	SDS(%)	RS(%)	RDS(%)	SDS(%)	RS(%)	RDS(%)	SDS(%)	RS(%)
0	74.7 ± 1.3	12.2 ± 0.5	13.1 ± 1.0	72.4 ± 0.8	10.7 ± 1.1	16.8 ± 0.2	69.9 ± 0.5	17.9 ± 1.5	12.2 ± 1.2
5	74.8 ± 0.8	12.0 ± 1.2	13.2 ± 0.9	76.7 ± 1.5	10.3 ± 0.2	13.0 ± 1.4	70.7 ± 1.1	10.7 ± 0.9	18.6 ± 1.0
120	79.0 ± 0.5	8.3 ± 0.6	12.7 ± 1.2	80.1 ± 1.2	7.7 ± 0.8	12.2 ± 0.7	73.8 ± 1.0	6.9 ± 0.6	19.3 ± 1.5
720	84.1 ± 1.1	4.0 ± 0.6	12.0 ± 0.3	89.0 ± 1.4	4.0 ± 0.7	7.1 ± 0.4	75.4 ± 1.6	4.8 ± 0.4	19.8 ± 0.7

*RDS* rapidly digestible starch, *SDS* slowly digestible starch, *RS* resistant starch.

### Structural model of acid degradation

**Fig. 6:**
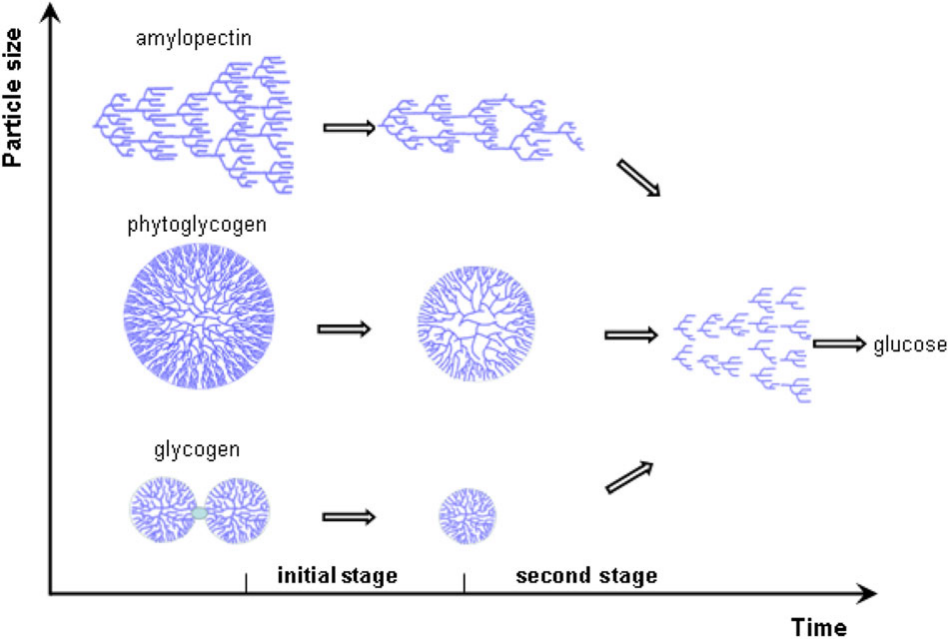
Structural models of glucose polymers subjected to acid degradation.

The structural change of phytoglycogen, amylopectin and glycogen subjected to acid degradation was deduced and the possible model is present in Fig. 6. According to the previous studies^4,13,16^, both phytoglycogen and amylopectin are the highly branched α-D-glucan naturally occurring in plants. The starch biosynthesis reactions are coordinately catalyzed by the combined action of starch synthase, branching enzyme and debranching enzyme to form amylose and amylopectin in the amyloplasts^1^. In the debranching enzyme-deficient mutant of sugary-1 endosperm, phytoglycogen is formed as an immature precursor of amylopectin with more highly branched structure. A mathematical model has been developed for describing the Whelan structure of phytoglycogen^38^. The nanoparticle has a spherical shape that is organized into concentric tiers or compact with a uniform density throughout the interior of the particles. Every B-chain is in the inner tier and every A-chain is in the outer tier. Each A- or B-chain has 12-14 glucose residues and there are a maximum of 12 tiers in the molecule, which means there is a maximum of about 53,000 glucose residues and 2,100 non-reducing ends. Within the tiered structure, the actual size is limited by the space available at the periphery for further branching. The differences in molecular architecture within the phytoglycogen led to a change in density change from the internal (950 g/mol·nm3) to external (1,600 g/mol·nm3) regions, different from the density of homogeneous amylopectin with approximately 60 g/mol·nm^3^^7,26^. Also, the external region of the phytoglycogen was reported to be about 3.3 nm thick. Although the branching can be thought of as producing tiers and generations within the dendritic molecule, the flexibility of the chains results in backfolding of the chains so that the tiers are not concentric, but instead they are intermingled with a uniform density throughout the molecule. Simmons et al. also reported that phytoglycogen nanoparticles were compact with a uniform density throughout the interior of the particle and had the short hairy chains that extend from the outer surface of the uniform particles with the core-coil geometry^10^. Moreover, glycogen is the intracellular major glucose reserves in the animal tissues, and has been identified to bear a resemblance to phytoglycogen from plant origin^12^. In the Krisman model, there is a protein core surrounded by several tiers of branch points, with crowding in the outer layers^39^. Glycogen comprises complex α particles made up of smaller β particles, ranging from 20 to 300 nm in diameter. The two mechanisms in glycogen synthesis for stopping chain growth were proposed: branching enzyme dominated chain growth in the innermost region of complex branched polymers with low ρ, while steric hindrance (crowding) dominated chain stoppage in the high-density outer region^40^. Also, the protein glycogenin was confirmed as binding agent joining β particles together into large α particles^32^. Overall, phytoglycogen or amylopectin appeared to be held together only by glycosidic linkages, whereas α-particles in an animal-based glycogen were held together by a combination of covalent and non-covalent links involving a protein.

In combination with the results of fine structure and particle radius distribution (Table 3 and Fig. 5), amylopectin with a higher particle radius and lower density was hydrolyzed faster than phytoglycogen or glycogen counterparts, suggesting a less branch and relatively flexible structure tends to give higher rate of hydrolysis degradation. In the initial stage of acid hydrolysis (approximately 0-30 min), an initial rapid reduction of molar mass with increasing time was observed, corresponding to the hydrolysis of less dense regions of glucose polymer, especially for amylopectin. Also, the results of particle radius distributions indicated that the protein in glycogen α particles was more acid-labile than the glycosidic linkage under mild conditions, resulting in the smaller β particles^15^. In the second stage, the more gradual reduction in molar mass at longer hydrolysis times was measured, depicting the non-preferential linkage cleavage as observed for the degradation course of the particles with denser regions. The similar dependence of the particle size on hydrolysis time was reported by Shamana and Dutcher^25^, revealing a reduction in the number-weighted hydrodynamic radius of the phytoglycogen from an initial value of 22 nm to 12 nm. The particle radius distribution of phytoglycogen decreased uniformly with hydrolysis time as shown in Fig. 5.

In this work, the particulate structure and properties of phytoglycogen, amylopectin and glycogen subjected to acid degradation was elucidated. The degree of hydrolysis was in the following order under the same reaction conditions: amylopectin > phytoglycogen > glycogen. Based on the data for the mathematical model of acid degradation reaction, the linkages changes of glucose polymer was related with the composition of chain segment as a function of the molar mass. Moreover, structural model for linkage scission of high-branched polymer was deduced to interpret the structure-property relationship, which would aid in the discovery and the development process for novel foods with tailor-made features for further miscellaneous applications.

## Methods

### Materials and reagents

Phytoglycogen was extracted from su-1 maize fresh kernel as described in a previous study^7^. The obtained phytoglycogen particle had a uniform average particle radius of 72.0 nm with more than 99 % phytoglycogen content and less than 1% of protein impurity. The type VI-B α-amylase from porcine pancreas (Cat. No. A3176, ≥5 units/mg solid), glycogen from oyster (Cat. No. G8751) and amylopectin from maize (Cat. No. 10120) were purchased from Sigma-Aldrich Chemical Co. (St. Louis, MO, USA). Amyloglucosidase from *Aspergillus niger* and the glucose oxidase-peroxidase assay kits were from Megazyme International Ireland Ltd. (Wicklow, Ireland). All reagent-grade chemicals were obtained from Sinopharm Chemical Reagent Co., Ltd. (Shanghai, China).

### Mild acid hydrolysis

Phytoglycogen, glycogen and amylopectin samples were completely dissolved in 1.0 M HCl solution (1.0 g sample/20 mL acid) and treated at 50 °C for different periods of time (0 min, 5 min, 30 min, 2 h, 6 h, 12 h, 24 h, 48 h, 72 h or 120 h) according to our previous work^18^. Aliquot was collected at different hydrolysis times and then neutralized and precipitated with three volumes of ethanol. The sample was centrifuged at 5000 g for 10 min and the residue was dried under vacuum oven. The degree of hydrolysis for degradation reaction was calculated as the percentage of the total carbohydrate from the supernatant at a given time to the initial dry weight of sample. Total carbohydrate was determined using the phenol-sulphuric acid method^14^.

### Molar mass distribution

The molar mass and radius of gyration were measured using the size-exclusion chromatography coupled with multi-angle laser light scattering and refractive index detector (SEC-MALLS-RI) system (Wyatt Technology, Santa Barbara, CA, USA) equipped with SB-805 and 80 tandem Shodex OH-pak columns (Showa Denko K.K., Tokyo, Japan) and a DAWN HELEOS II laser photometer fitted with a He-Ne laser (λ = 632.8 nm) as well as an OPTILAB^®^ T-rEX Interferometric Refractometer. For the measurement, 10 mg of each sample were dispersed in 1 mL of deionized water (prefiltered by 0.22-μm nylon syringe filters) and heated in a boiling-water bath for 10 min. The flow rate of distilled water as mobile phase was set at 0.5 mL/min. A value of 0.138 was used as *dn/dc* for molar weight calculation^4^, and the weight-average molar mass (Mw), number-average molar mass (Mn), z-average molar mass (Mz) and z-root mean square radius of gyration (Rz) were obtained using the first order Berry model with the Version 5.3.4.14 ASTRA software^10^. The molecular density (ρ) was calculated as Mw/Rz^3^ ^7^.

The kinetics of degradation reaction was proposed as follows: $\frac{{\rm{dB}}}{{\rm{dt}}}=-\frac{1}{{\rm{Mn}}}\frac{{\rm{dMn}}}{{\rm{dt}}}={\rm{k}}{(\frac{{\rm{Mn}}}{162})}^{\alpha }$ (1), in which B was the total number of broken linkages, k was the rate constant (1/s), Mn was number-average molar mass of polymer (g/mol), 162 was the molar mass of monomer unit (g/mol), and α was the order of reaction^29^. Our data were rationalized as the depolymerization process, and the simplified equation of $\frac{1}{Mn{(t)}^{\alpha }}-\frac{1}{Mn{(0)}^{\alpha }}=\alpha \frac{k}{{162}^{\alpha }}t$(2) was used to calculate the experimental kinetics of acid degradation, where Mn(0) was the initial Mn, and Mn(t) was the corresponding value after the time t.

### Hydrodynamic radius measurement

The triplicate measurement was conducted with a Zetasizer Nano ZS particle size analyzer (Malvern Instruments Ltd., Malvern, UK) using dynamic light scattering (DLS). The sample was added to distilled water at a concentration of 0.01% (w/w) and boiled at 90 °C with stirring for 15 min to completely dissolve before measurement. The intensity-averaged radius value measured using DLS was skewed to larger values, which was roughly twice that of number-averaged values, whereas the number-weighted particle radius was consistent with the measured result from other techniques, such as atomic force microscopy and neutron scattering^25^. Thus, the number-averaged hydrodynamic radius was determined using the CONTIN algorithm within the Malvern software^41^. All the tests were performed at least in duplicate.

### Proton nuclear magnetic resonance spectroscopy (^1^H NMR)

^1^H NMR spectra were recorded using an AVANCE III Digital NMR spectrometer (Brucker Co., Billerica, MA) operating at 500 MHz and 80 °C. Sample was exchanged with deuterium in three rounds of lyophilization with 99.99% deuterium oxide and then dissolved in 0.45 mL 99.99% deuterium oxide. Using version 8.0 MestReNova software, percentages of glycosidic bond were determined using the area ratios in which peaks approximately at 5.4 and 5.0 ppm were assigned to protons of α-1,4 and α-1, 6 linked units, respectively.

### Simulated gastrointestinal digestion

The in-vitro digestion for glucose release was analysed according to the Englyst approach with a slight modification^42^. Sample (200 mg) was dissolved in 4 mL of phosphate buffer (0.2 M, pH 5.2). After the solution was kept boiling bath 30 min and equilibrated at 37 °C for 5 min, seven glass balls (10 mm diameter) and enzyme preparation (5.0 ml of 290 U/mL α-amylase and 6 U/mL amyloglucisidase) were added. Then, the samples were shaken in a 37 °C water bath at 150 rpm. Aliquots of hydrolysed solution (0.05 mL) were taken at two time points: 20 and 120 min, and mixed with 1 mL of 90% ethanol (v/v) to deactivate the enzymes. The glucose content was determined using the glucose oxidase/peroxidase assay kit. The percentages of rapidly digestible starch (RDS), slowly digestible starch (SDS) and resistant starch (RS) were calculated using the formulas^4^: RDS (%) = (G120 – FG) ×0.9×100 (3), SDS (%) = (G120 – G20) ×0.9×100 (4), RS (%) = (TG – FG) ×0.9×100 – RDS – SDS (5), in which G20 is released glucose after 20 min, G120 was released glucose after 120 min, FG was free glucose, and TG was total glucose.

### Statistical analysis

Experiments were carried out in triplicate and all data were expressed as the mean values ± standard deviations. Origin 8.5 (OriginLab Inc., USA) was employed for statistical analysis. A level of 0.05 was set to determine statistical significance.

### Reporting summary

Further information on research design is available in the Nature Portfolio Reporting Summary linked to this article.

## Supplementary information

Reporting summary

## Data Availability

The datasets generated during and/or analyzed during the current study are available from the corresponding author on reasonable request.

## References

[1] BeMiller, J. N., & Whistler, R. L. *Starch: Chemistry and technology* (Third Edition). New York: Academic Press (2009).

[2] Miao M, Jiang B, Jin Z, BeMiller JN (2018). Microbial starch-converting enzymes: Recent insights and perspectives. Compr. Rev. Food Sci. Food Saf..

[3] Miao M, BeMiller JN (2023). Enzymatic approaches for structuring starch to improve functionality. Annu. Rev. Food Sci. Technol..

[4] Miao M (2014). Structure and digestibility of endosperm water-soluble α-glucans from different sugary maize mutants. Food Chem..

[5] Besford QA, Cavalieri F, Caruso F (2020). Glycogen as a building block for advanced biological materials. Adv. Mater..

[6] Xue J, Luo Y (2021). Properties and applications of natural dendritic nanostructures: Phytoglycogen and its derivatives. Trends Food Sci. Technol..

[7] Miao M, Li R, Huang C, Jiang B, Zhang T (2015). Impact of β-amylase degradation on properties of sugary maize soluble starch particles. Food Chem..

[8] Bezborodkina NN, Chestnova AY, Vorobev ML, Kudryavtsev BN (2018). Spatial structure of glycogen molecules in cells. Biochemistry.

[9] Kim D, Duhamel J (2023). Interior of glycogen probed by pyrene excimer fluorescence. Carbohydr. Polym..

[10] Simmons J (2020). Structure, hydration, and interactions of native and hydrophobically modified phytoglycogen nanoparticles. Biomacromolecules.

[11] Zhang P (2018). Exploring glycogen biosynthesis through Monte Carlo simulation. Int. J. Biol. Macromol.

[12] Sullivan MA (2012). Molecular insights into glycogen α-particle formation. Biomacromolecules.

[13] Shi Y, Ye F, Chen Y, Hui Q, Miao M (2020). Dendrimer-like glucan nanoparticulate system improves the solubility and cellular antioxidant activity of coenzyme Q10. Food Chem..

[14] Shi Y, Ye F, Lu K, Hui Q, Miao M (2020). Characterizations and bioavailability of dendrimer-like glucan nanoparticulate system containing resveratrol. J. Agric. Food Chem..

[15] Xie Y, Yao Y (2018). Octenylsuccinate hydroxypropyl phytoglycogen, a dendrimer-like biopolymer, solubilizes poorly water-soluble active pharmaceutical ingredients. Carbohydr. Polym..

[16] Ye F (2018). Characterisations of oil-in-water Pickering emulsion stabilized hydrophobic phytoglycogen nanoparticles. Food Hydrocoll..

[17] Karlsson A, Singh SK (1999). Acid hydrolysis of sulphated polysaccharides. Desulphation and the effect on molecular mass. Carbohydr. Polym..

[18] Miao M, Jiang B, Zhang T, Jin Z, Mu W (2011). Impact of mild acid hydrolysis on structure and digestion properties of waxy maize starch. Food Chem..

[19] Powell PO (2015). Acid hydrolysis and molecular density of phytoglycogen and liver glycogen helps understand the bonding in glycogen α (composite) particles. PLoS ONE.

[20] Li C, Hu Y (2021). Effects of acid hydrolysis on the evolution of starch fine molecular structures and gelatinization properties. Food Chem..

[21] Ulbrich M, Daler JM, Flöter E (2020). Acid hydrolysis of corn starch genotypes. II. Impact on functional properties. Food Hydrocoll..

[22] Wang S, Blazek J, Gilbert E, Copeland L (2012). New insights on the mechanism of acid degradation of pea starch. Carbohydr. Polym..

[23] de la Concha BBS (2018). Acid hydrolysis of waxy starches with different granule size for nanocrystal production. J. Cereal Sci..

[24] Mao H (2021). Structural comparisons of pyrodextrins during thermal degradation process: The role of hydrochloric acid. Food Chem..

[25] Shamana H, Dutcher JR (2022). Transition in the glassy dynamics of melts of acid- hydrolyzed phytoglycogen nanoparticles. Biomacromolecules.

[26] Huang L, Yao Y (2011). Particulate structure of phytoglycogen nanoparticles probed using amyloglucosidase. Carbohydr. Polym..

[27] Powell PO (2014). Extraction, isolation and characterisation of phytoglycogen from su-1 maize leaves and grain. Carbohydr. Polym..

[28] Prats C, Graham TE, Shearer J (2018). The dynamic life of the glycogen granule. J. Biol. Chem..

[29] Basedow AM, Ebert KH, Ederer HJ (1978). Kinetic studies on the acid hydrolysis of dextran. Macromolecules.

[30] Ball SG, Morell MK (2003). From bacterial glycogen to starch: Understanding the biogenesis of the plant starch granule. Annu. Rev. Plant Biol..

[31] Yun S-H, Matheson NK (1993). Structures of the amylopectins of waxy, normal, amylose-extender, and wx ae genotypes and of the phytoglycogen of maize. Carbohydr. Res..

[32] Tan X (2018). Proteomic investigation of the binding agent between liver glycogen β particles. ACS Omega.

[33] Miao M, Jiang B, Zhang T (2009). Effect of pullulanase debranching and recrystallization on structure and digestibility of waxy maize starch. Carbohydr. Polym..

[34] Miao M, Hamaker BR (2021). Food matrix effects for modulating starch bioavailability. Ann. Rev. Food Sci. Technol..

[35] Pazur JH, Ando T (1960). The hydrolysis of glucosyl oligosaccharides with α-D-(1,4) and α-D-(1,6) bonds by fungal amyloglucosidase. J. Biol. Chem..

[36] Zhou X, Campanella OH, Hamaker BR, Miao M (2021). Deciphering molecular interaction and digestibility in retrogradation of amylopectin gel network. Food Function.

[37] Zhang G, Ao Z, Hamaker BR (2008). Nutritional property of endosperm starches from maize mutants: A parabolic relationship between slowly digestible starch and amylopectin fine structure. J. Agric. Food Chem..

[38] Melendezhevia E, Waddell TG, Shelton ED (1993). Optimization of molecular design in the evolution of metabolism:The glycogen molecule. Biochem. J..

[39] Goldsmith E, Sprang S, Fletterick R (1982). Structure of maltoheptaose by difference Fourier methods and a model for glycogen. J. Mol. Biol..

[40] Deng B (2015). The mechanism for stopping chain and total-molecule growth in complex branched polymers, exemplified by glycogen. Biomacromolecules.

[41] Roman L (2022). Changes to fine structure, size and mechanical modulus of phytoglycogen nanoparticles subjected to high-shear extrusion. Carbohydr. Polym..

[42] Englyst HN, Kingman SM, Cummings JH (1992). Classification and measurement of nutritionally important starch fractions. Eur. J. Clin. Nutr..

